# Commensal-derived metabolites govern *Vibrio cholerae* pathogenesis in host intestine

**DOI:** 10.1186/s40168-019-0746-y

**Published:** 2019-09-14

**Authors:** Jin Sun You, Ji Hyun Yong, Gwang Hee Kim, Sungmin Moon, Ki Taek Nam, Ji Hwan Ryu, Mi Young Yoon, Sang Sun Yoon

**Affiliations:** 1 Department of Microbiology and Immunology, Yonsei University College of Medicine, 50-1 Yonsei-ro, Seodaemun-gu Seoul, Seoul, 03722 Korea; 2 Brain Korea 21 PLUS Project for Medical Sciences, Yonsei University College of Medicine, Seoul, 03722 Korea; 3 Severance Biomedical Science Institute, Yonsei University College of Medicine, Seoul, 03722 Korea; 4 Institute for Immunology and Immunological Diseases, Yonsei University College of Medicine, Seoul, 03722 Korea

**Keywords:** *Vibrio cholerae*, Gut microbiota, Colonization resistance, Short-chain fatty acids, Metabolomics, *Bacteroides vulgatus*, Clindamycin, Amino sugars

## Abstract

**Background:**

Recent evidence suggests that the commensal microbes act as a barrier against invading pathogens and enteric infections are the consequences of multi-layered interactions among commensals, pathogens, and the host intestinal tissue. However, it remains unclear how perturbations of the gut microbiota compromise host infection resistance, especially through changes at species and metabolite levels.

**Results:**

Here, we illustrate how *Bacteroides vulgatus*, a dominant species of the Bacteroidetes phylum in mouse intestine, suppresses infection by *Vibrio cholerae*, an important human pathogen. Clindamycin (CL) is an antibiotic that selectively kills anaerobic bacteria, and accordingly Bacteroidetes are completely eradicated from CL-treated mouse intestines. The Bacteroidetes-depleted adult mice developed severe cholera-like symptoms, when infected with *V*. *cholerae*. Germ-free mice mono-associated with *B*. *vulgatus* became resistant to *V*. *cholerae* infection. Levels of *V*. *cholerae* growth-inhibitory metabolites including short-chain fatty acids plummeted upon CL treatment, while levels of compounds that enhance *V*. *cholerae* proliferation were elevated. Furthermore, the intestinal colonization process of *V*. *cholerae* was well-simulated in CL-treated adult mice.

**Conclusions:**

Overall, we provide insights into how a symbiotic microbe and a pathogenic intruder interact inside host intestine. We identified *B*. *vulgatus* as an indigenous microbial species that can suppress intestinal infection. Our results also demonstrate that commensal-derived metabolites are a critical determinant for host resistance against *V*. *cholerae* infection, and that CL pretreatment of adult mice generates a simple yet useful model of cholera infection.

## Background

*Vibrio cholerae* is the causative agent of pandemic diarrheal disease, cholera*.* While cholera toxin (CT) and toxin-coregulated pilus (TCP) are known to be the major virulence determinants, its pathogenic mechanisms are starting to be understood as consequences of interaction with indigenous microbes, collectively termed gut microbiota [1–4]. A key feature of the gut microbiota is its protective capacity against enteropathogenic infections, termed “colonization resistance” [5–7]. This property can be ascribed to the microbial ecosystem that is formed within the host intestine. This microbial ecosystem is an ever-changing community of various microbial species regulated by a complex network of microbiota-intrinsic and microbiota-extrinsic factors [8].

Microbiota-intrinsic factors such as interactions between the gut-residing species serve as the primary determinant of community composition. Such interactions include interbacterial niche competition [9] and secretion of antimicrobial substances [10]. For example, *Bacteroides* species that reside in the human gut, such as *Bacteroides fragilis and Bacteroides uniformis*, utilise membrane attack complex/perforin toxins BSAP-1 and BSAP-2 for intraspecies antagonism [11]. Microbiota-extrinsic factors include inflammation, diet, and antibiotic treatment. However, the aforementioned microbiota-intrinsic and extrinsic factors do not operate in exclusion of another. For example, compositional shift induced by an external factor may, in turn, modulate the host immune response via production of specific metabolites [8].

Recent studies have demonstrated that antibiotic treatments alter the gut microbiota in humans and other mammals [12–15], and increase the susceptibility of the host to infections by various enteric pathogens such as *Shigella flexneri* [16], *Salmonella enterica* [17], *Clostridium difficile* [18, 19], and vancomycin-resistant *Enterococcus* [20]. The abolishing effect of antibiotic treatment on host resistance to infection is most likely implemented through a multitude of factors, including suppression of specific microbial species, alteration of the metabolomic landscape as a result of the changed microbiome composition, and/or host responses [21–23]. These findings prompted us to investigate the effects of different classes of antibiotics on the gut microbiota and how they relate to host resistance against *V*. *cholerae* infection.

In this study, we uncover a unique microbiota-extrinsic treatment that induces severe cholera-like symptoms in adult mice, that are otherwise completely resistant. Furthermore, we show that a dramatic shift in metabolome production profile accounts for the compromised infection resistance in the host. This report highlights the importance of commensal-derived metabolites as a crucial determinant of host susceptibility to enteric infection.

## Results

### Clindamycin-treated adult mice exhibited dramatic gut microbiota compositional changes and became susceptible to *V*. *cholerae* infection

**Fig. 1:**
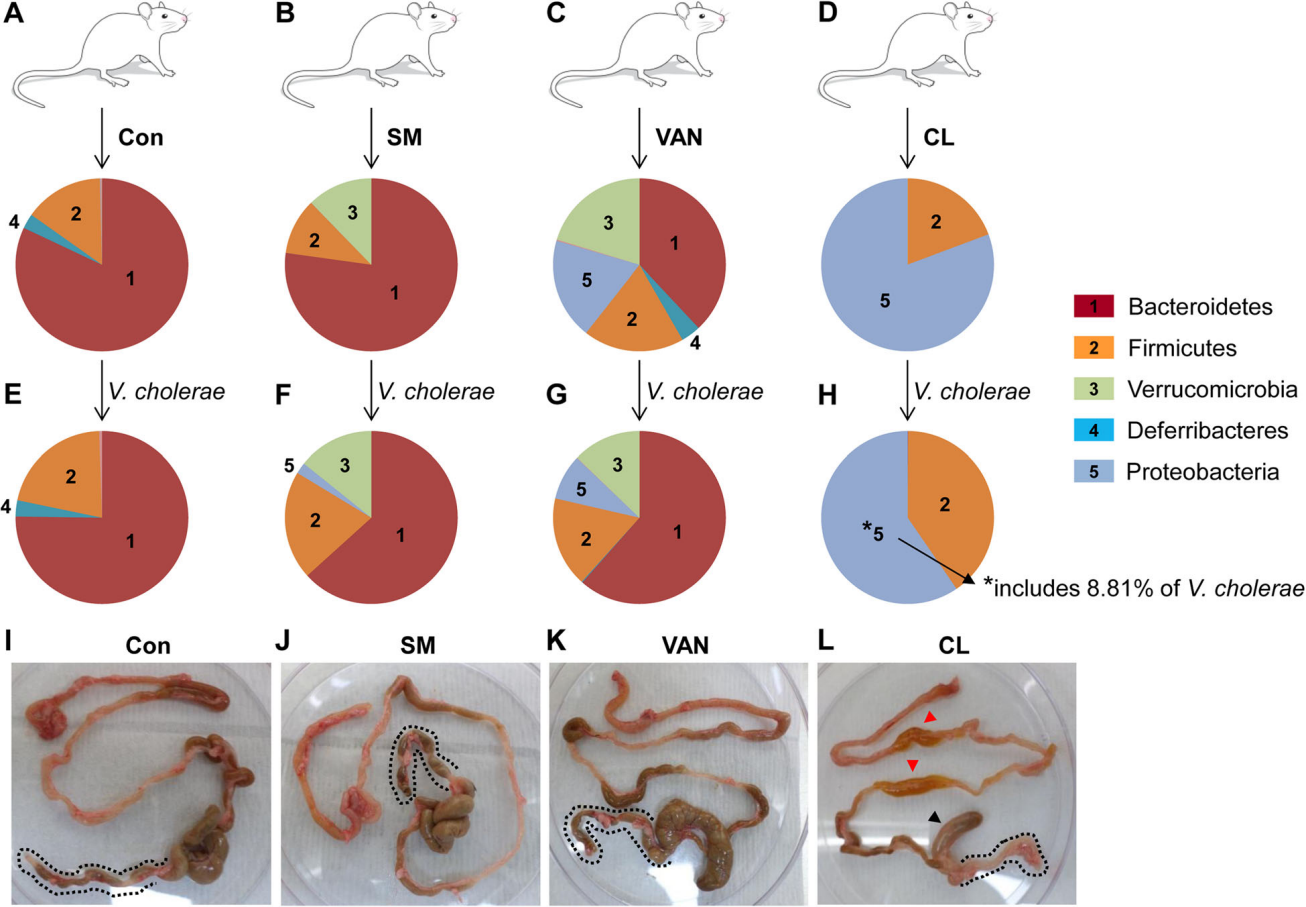
Microbial population changes in response to the antibiotic treatments and host responses to *V*. *cholerae* infection in each treatment group. Adult mice were divided into four groups and treated with PBS (**a**), streptomycin (SM) (**b**), Vancomycin (VAN) (**c**), or clindamycin (CL) (**d**). Treatments were performed as described in “Methods” section. Fecal matter was collected at the end of the treatment, and microbial DNA was extracted for 16S rRNA gene amplicon sequencing. Microbial compositions at the phylum level are shown in pie charts. Relative abundance of each phylum is proportional to the arc length of each slice. Five dominant phyla (i.e., Bacteroidetes, Firmicutes, Verrucomicrobia, Deferribacteres, and Proteobacteria) numbered from 1 to 5 are indicated with different colors. Following treatment, mice in each group were infected with 5 × 10^8^ CFU of N16961 cells. At 24 h post-infection, fecal matter collected from each group (**e**–**h**) was subject to microbial composition analysis. *The Proteobacteria phylum shown in panel H includes *V*. *cholerae* at 8.81% abundance. At the end of the infection period, mouse gastrointestinal tracks were extracted from each group to visualize infection-induced phenotypes (**i**–**l**). Red arrowheads shown in panel **l** indicate regions of small intestine with fluid accumulation, while a black arrowhead shows the cecum, where the size is remarkably smaller than in other mice. Dotted lines in panels **i**–**l** indicate the large intestines

Colonization of enteric pathogens occurs depending on the composition of indigenous microbes inside the host intestine [5, 24, 25]. In order to observe the effects of gut microbiota compositional changes on host infection resistance under diverse experimental conditions, we treated adult C57BL/6 specific pathogen-free (SPF) mice (8 weeks of age) with three different antibiotics that have a distinct mode of bacterial killing; streptomycin (SM), vancomycin (VAN), and clindamycin (CL). SM is a broad-spectrum antibiotic that targets both Gram-positive and Gram-negative bacteria. VAN is an effective antibiotic against Gram-positives [26], while CL is known to selectively kill anaerobes [27, 28]. After daily treatment for 5 days, mouse feces were collected to analyze microbiota composition by 16S rRNA gene sequencing. Five bacterial phyla were found to have characteristic distributions among groups (Fig. 1a–d). In the untreated control group, Bacteroidetes was the most abundant phylum and the members of that single phylum occupied approximately 80% of the entire microbiota population (Fig. 1a). Upon SM treatment, the Verrucomicrobia phylum emerged, while the Bacteroidetes phylum maintained its predominant occupancy (Fig. 1b). VAN treatment resulted in multiplication of bacterial cells belonging to the Verrucomicrobia and Proteobacteria phyla, both of which are Gram-negatives (Fig. 1c). As a result of CL treatment, the Bacteroidetes phylum was eradicated, while the Firmicutes phylum remained largely unchanged in number (Fig. 1d). This observation is consistent with previous findings that CL is effective in treating infections of *Bacteroides fragilis*, a major species of the Bacteroidetes phylum [29, 30]. On the other hand, the Proteobacteria phylum underwent explosive expansion during CL treatment, further demonstrating that CL is not effective in inhibiting the growth of facultative anaerobes (Fig. 1d).

Subsequently, the mice of each antibiotic group were infected with 5 × 10^8^ CFU of the *V*. *cholerae* 7th pandemic strain, N16961, to monitor susceptibility to infection and infection-induced microbiota composition changes. In the control and SM-treated groups, microbiota composition did not change much following *V*. *cholerae* infection (Fig. 1e, f). In response to N16961 infection, the relative abundance of the Bacteroidetes phylum increased in the VAN-pretreated group (Fig. 1g vs. c). In CL-treated mice, the relative abundance of the Firmicutes phylum increased, while that of the Proteobacteria phylum decreased in response to *V*. *cholerae* infection (Fig. 1h). Importantly, among the Proteobacteria phylum detected in Fig. 1h, ~ 8.81% was found to be derived from *V*. *cholerae*-specific 16S rRNA gene sequence (Fig. 3). This result is worthy of particular attention because *V*. *cholerae*-specific sequence was not detected in the Proteobacteria populations in other groups (Fig. 1f, g).

Our results indicate that *V*. *cholerae* may actively colonize the adult mouse intestine under conditions created by CL treatment. To gain more insight into the outcomes of *V*. *cholerae* infection, we investigated whether any anatomical change was induced in the gastrointestinal tract ranging from the small intestine to the rectum. In the first three experimental groups, mouse tissues appeared similar to one another and no major changes were observed following *V*. *cholerae* infection (Fig. 1i–k). In contrast, infection-induced phenotypes were clearly demonstrated in the CL-treated group for the following indications (Fig. 1l). First, fluid accumulation was clearly observed in the small intestine (red arrowheads). Second, the size of the cecum was markedly smaller compared to those in other groups (black arrowhead). Third, the colon appeared shorter and more interestingly, became transparent, while the colons in other groups were filled with feces (dotted line around the colon). Forth, fecal matter discharged from the CL-treated group was highly aqueous (data not shown). Together, these results demonstrate that *V*. *cholerae* infection induces cholera-like symptoms in adult mice, when their gut microbiota compositions are altered by CL treatment.

### CL treatment with a different regimen similarly induced hypersusceptibility to *V*. *cholerae* infection

Our results, shown in Fig. 1, demonstrate that symptoms of watery diarrhea observed in human cholera patients can also be induced in adult mice by a simple antibiotic pretreatment. In the above experiment, mice were treated with a mild concentration of CL daily for 5 days. To elucidate whether CL treatment could elicit similar effects within a shorter period, we conducted another set of infection experiments with a single treatment of higher CL dosage (Additional file 1: Fig. S1A). Similar to previous results with lower CL dosage, significantly increased fluid accumulation (FA) was induced in adult mouse intestine at 24 h post-infection. The FA ratio was determined to be ~ 0.176, a value ~ 1.6-fold higher in comparison to that of the uninfected group (Additional file 1: Fig. S1B). Consistent with this finding, robust *V*. *cholerae* colonization was observed in CL-pretreated mice. The number of *V*. *cholerae* cells recovered from the small intestine (SI) or cecum was remarkably larger in CL-treated vs. control group. *V*. *cholerae* cells of > 10^9^ CFU were grown per gram sample in SI and cecum, respectively. Likewise, ~ 10^9^ CFU was also detected in fecal pellets shed from CL-treated mice (Additional file 1: Fig. S1C). In contrast, *V*. *cholerae* cells were not detected at all in feces discharged from the control group. Given that an infection dosage of 5 × 10^8^ CFU was used, these results demonstrate that (i) *V*. *cholerae* cells were almost completely eliminated inside the intestine of regular adult mice while (ii) the same number of inoculated cells was more highly proliferated in the CL-pretreated adult mouse intestine.

### Germ-free mice transplanted with feces of CL-treated mice were susceptible to *V*. *cholerae* infection

**Fig. 2:**
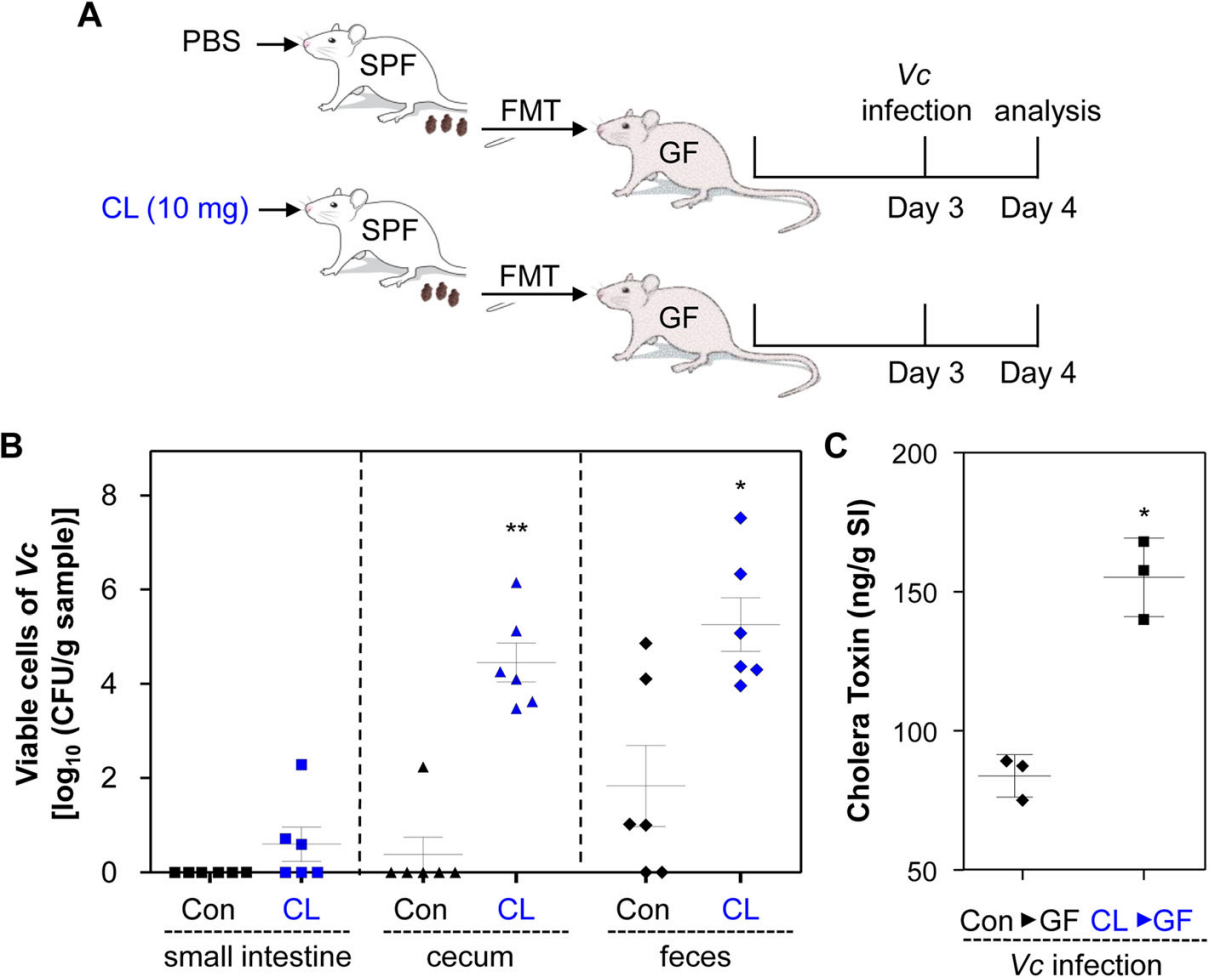
Germ-free mice transplanted with feces of CL-treated mice are susceptible to *V*. *cholerae* infection. **a** Schematic diagram of the experimental procedure. Germ-free mice (C57BL/6, 8~9 weeks old, *n* = 6 per group) received fecal suspensions (50 μL) derived from PBS- or CL-treated SPF mice. Following 3 days of stabilization, transplanted mice were challenged with *V*. *cholerae* infection (5 × 10^8^ CFU) for 24 h. **b** The SI and cecum were removed from each mouse for tissue homogenization. Fresh fecal matter was collected to prepare fecal suspensions. Aliquots of lysates or suspensions were serially diluted to count CFU of *V*. *cholerae* on LB agar supplemented with 200 μg/mL SM. Values are displayed on a log scale as mean ± SEM for each group. *P < 0.05 versus bacterial CFU detected in the control group. **P < 0.001 versus bacterial CFUs detected in the control group. **c** In a separate set of experiments (*n* = 3 per group), small intestines were extracted for tissue homogenization, and aliquots of homogenates were assessed for detection of CT by ELISA. *P < 0.01 versus CT level in the control group

Previous studies have revealed that CL treatment can modulate the production of pro-inflammatory cytokines, especially in response to lipopolysaccharide [31–33]. Furthermore, CL at sub-lethal concentrations stimulate neutrophil phagocytosis in vitro against *Staphylococcus aureus* [34] and *Bacteroides* spp. [35]. While our results strongly suggest that CL-induced changes in gut microbiota composition are associated with elevated susceptibility to *V*. *cholerae* infection, these previous reports also indicate that enhanced *V*. *cholerae* infectivity in adult mice might be attributed to CL-induced modulation of the host immune response. To address this issue, we conducted fecal microbiota transplantation (FMT) to germ-free (GF) mice and tested whether the GF mice with reconstituted intestinal microbiota develop differential resistance to *V*. *cholerae* infection. Fecal pellets freshly collected from PBS- or CL-treated mice were resuspended in PBS and transferred to GF mice via oral gavage. Following a 3-day transplant stabilization period, 5 × 10^8^ N16961 cells were infected (Fig. 2a). GF mice transplanted with PBS-treated control fecal pellets developed strong resistance to the infection. Viable *V*. *cholerae* was recovered neither from the SI of all six mice nor from the cecum isolated from five out of six mice (Fig. 2b). While varying degrees of N16961 cells were detected in feces discharged from the GF mice transplanted with control feces, four out of six mice produced feces that contained ten or fewer N16961 cells per gram (Fig. 2b). In contrast, N16961 colonization occurred more actively in the GF mice transplanted with CL-treated and therefore Bacteroidetes-depleted feces. In cecum and feces, > 10^4^-fold and > 10^3.4^-fold higher bacterial colonization were observed, respectively, compared with the control group (Fig. 2b).

We next asked whether production of CT, a critical virulence determinant, was accordingly increased during active *V*. *cholerae* colonization. As shown in Fig. 2c, in response to *V*. *cholerae* infection, CT production was significantly increased in mice transplanted with fecal suspensions of CL-treated mice. Since CL would not be much left in the feces discharged from the CL-treated mice, our results demonstrate that altered gut microbiota composition induced by CL treatment is directly responsible for rendering the murine host more susceptible to *V*. *cholerae* infection.

### *Bacteroides vulgatus* is the predominant species in the Bacteroidetes phylum and it inhibited *V*. *cholerae* growth *in vivo*

Based on our results presented in Fig. 1, elevated *V*. *cholerae* infection in CL-pretreated mice could be ascribed either to the depletion of the Bacteroidetes or to the expansion of Proteobacteria phylum. Meanwhile, we noticed the followings. First, the Proteobacteria population similarly sufficiently propagated in VAN-treated mice, a group that did not develop cholera-like symptoms (Fig. 1c). Second, the relative abundance of the Bacteroidetes phylum increased from ~ 38% (before infection) to ~ 61% (after infection) in VAN-treated mice (Fig. 1c, g). Based on these results, we postulated that the lack of cholera-like symptoms in the VAN-treated group was due not only to the presence of the Bacteroidetes phylum but also to the infection-induced expansion of its population. We hypothesized that the Bacteroidetes phylum, which predominantly occupies the adult mouse intestine, plays a critical role in protecting its host from *V*. *cholerae* infection.

**Fig. 3:**
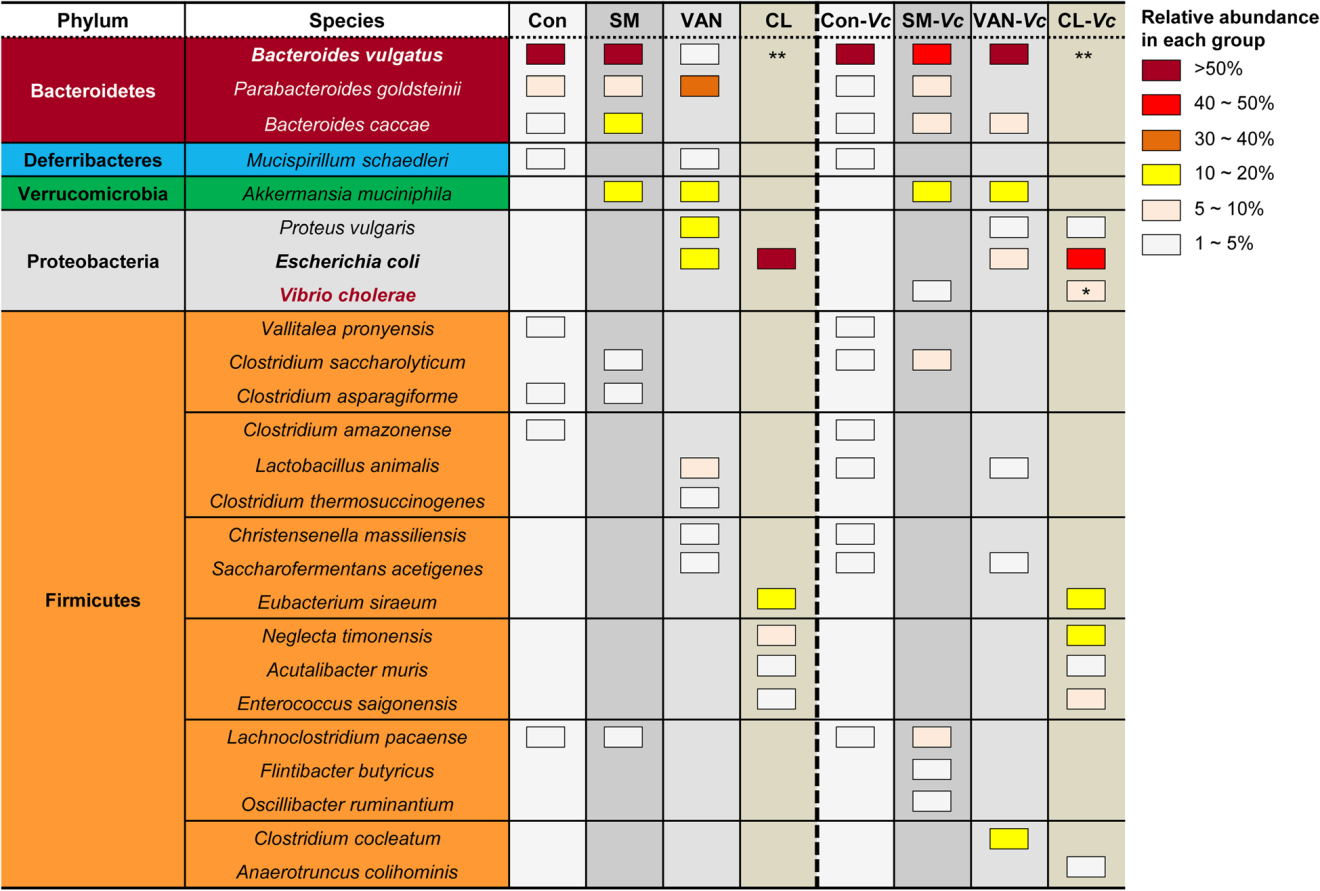
Species-level microbiota populations in response to antibiotic treatments and after *V*. *cholerae* infection. Distribution of commensal microbial species at the phylum (far-left column) and species (second column) levels are presented by colors in 8 different experimental conditions. Con, SM, VAN, and CL in the first low indicate microbial samples of corresponding antibiotic treatments and Con-*Vc*, SM-*Vc*, VAN-*Vc*, and CL-*Vc* indicate samples of the same groups following *V*. *cholerae* infection. Three species were determined to belong to Bacteroidetes (brown) and Proteobacteria (gray) phyla and 17 species belonging to the Firmicutes (orange) phylum were identified. Only bacterial species with a relative abundance of > % of the total microbiota population were selected and displayed. Relative abundance of each species is indicated by color-coded boxes. For example, brown boxes mean that the abundance of a given species under a specific condition is > 50%. A complete elimination of *Bacteroides vulgatus* is indicated with **, while a pink box denoted by * indicates 8.81% *V*. *cholerae* occupancy

To provide an insight into which bacterial species are core members of the Bacteroidetes phylum, we performed a species-level population analysis using 16S rRNA gene sequencing. When necessary, we conducted PCR reactions with species-specific primer sets and verified the sequences of amplification products (data not shown). Three distinct bacterial species were determined to belong to the Bacteroidetes phylum; *Bacteroides vulgatus*, *Parabacteroides goldsteinii*, and *Bacteroides caccae*. In control mice, *B*. *vulgatus* was found to be present in the largest quantity, with a relative abundance greater than 50% of the entire population (Fig. 3). In response to VAN treatment, *P*. *goldsteinii* propagated, whereas *B*. *vulgatus* was diminished in population size. Of note, however, the relative abundance of the *B*. *vulgatus* population was recovered to its original level following *V*. *cholerae* infection. *P*. *goldsteinii* has been reclassified from *Bacteroides goldsteinii* [36] and a strain isolated from human intestine was resistant to VAN treatment [37]. As shown in Fig. 1d, l, no 16S rRNA gene sequences matching any of these three species were detected in feces discharged from CL-treated mice either before or after *V*. *cholerae* infection (Fig. 3).

**Fig. 4:**
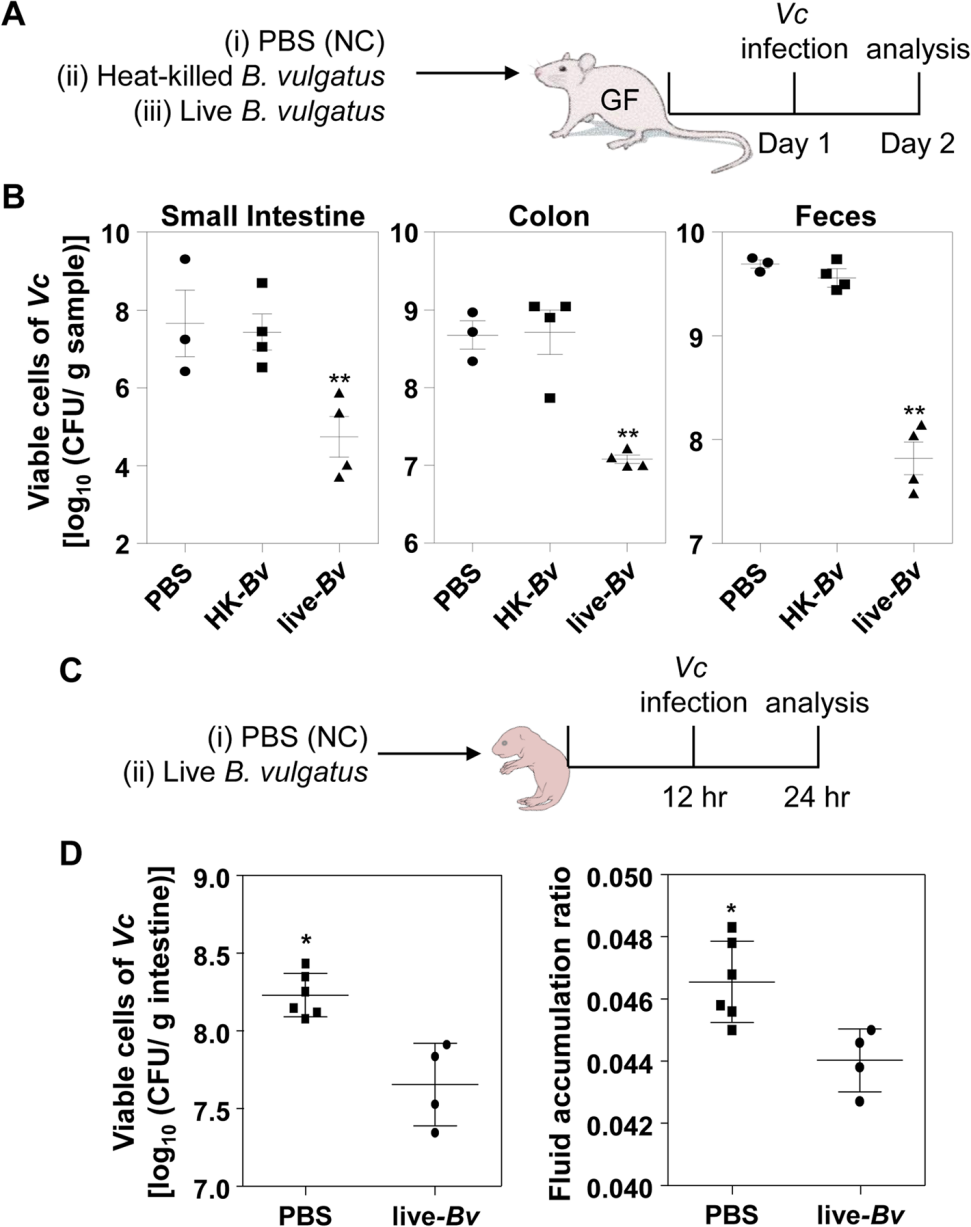
*B*. *vulgatus* inhibits *V*. *cholerae* growth in vivo. **a** Schematic diagram of the experimental procedure. Germ-free mice (C57BL/6, 8~9 weeks old, *n* = 3 or 4 per group) ingested 50 μL PBS (*n* = 3), heat-inactivated (autoclaved) *B*. *vulgatus* cell suspension (*n* = 4), or live *B*. *vulgatus* cell suspension (*n* = 4). The *B*. *vulgatus* cell suspensions contained 2 × 10^9^ CFU/mL. At 24 h post-association, mice were challenged with *V*. *cholerae* infection (5 × 10^8^ CFU) for 24 h. **b** Viable *V*. *cholerae* cells in SI, colon, or fecal pellets were enumerated and presented in log-scale. **P < 0.001 versus bacterial CFU in the control and heat-inactivated *Bv* groups. **c** Schematic diagram of the experimental procedure. Five-day-old infant mice (*n* = 4 or 6 per group) ingested 50 μL PBS (*n* = 6) or *B*. *vulgatus* cell suspension (*n* = 4). The *B*. *vulgatus* cell suspensions contained 1 × 10^9^ CFU/mL. At 12 h post-association, mice were challenged with *V*. *cholerae* infection (1 × 10^8^ CFU) for 12 h. **d** Viable *V*. *cholerae* cells in intestinal extract were enumerated and presented in log-scale. **P < 0.05 versus bacterial CFU in the control group. At the end of the infection period, fluid accumulation ratio was calculated by the equation of (intestine weight)/[(total body weight)-(intestine weight)]. *P < 0.05 versus the control group

Because *B*. *vulgatus* was the most dominant species in the adult mouse intestine, we next assessed, under a more defined condition, the effect of *B*. *vulgatus* on altering host susceptibility to *V*. *cholerae* infection. To this end, we mono-associated GF mice with a suspension of *B*. *vulgatus* cells, grown anaerobically in vitro (Fig. 4a). *B*. *vulgatus* colonies cultivated straight from mouse fecal suspensions were subject to RAPD assay and an identical amplification pattern was observed between isolated colonies, thereby leading us to conclude that most colonies were clonal (Additional file 2: Figure S2). As shown in Fig. 4a, GF mice were also mono-associated with heat-killed *B. vulgatus* before being infected with *V*. *cholerae* cells as an additional negative control. At 24 h post-infection, significantly reduced numbers of *V*. *cholerae* cells were recovered in animals that were mono-associated with live *B*. *vulgatus*. In SI and colon, ~ 830-fold and ~ 40-fold less *V*. *cholerae* colonization were observed, respectively, as compared with the PBS control group. Likewise, *V*. *cholerae* cell numbers, recovered from feces of the GF mice transplanted with live *B*. *vulgatus*, was ~ 75-fold lower than the PBS control (Fig. 4b). Importantly, *V*. *cholerae* colonization was never decreased in the animals mono-associated with heat-inactivated *B*. *vulgatus* cells (Fig. 4b). These results suggest that active *B*. *vulgatus* cells, when present in adult mouse intestine, can suppress *V*. *cholerae* colonization.

We next explored whether *B*. *vulgatus* also effectively inhibits *V*. *cholerae* colonization in the infant mouse model that has been used to study *V*. *cholerae* infection [38, 39]. Five-day-old infant mice were divided into two groups, with the first group being transplanted with *B*. *vulgatus* and the other group serving as a negative control (Fig. 4c). *B*. *vulgatus*, when transplanted in the intestine of the infant mouse, can also effectively suppress *V*. *cholerae* colonization and fluid accumulation (Fig. 4d). *V*. *cholerae* colonization was decreased ~ 4.47-fold in the presence of transplanted *B*. *vulgatus.* Although the difference between these two groups was less pronounced than what was observed in experiments with adult mice, *V*. *cholerae* colonization and infection-induced fluid accumulation were both reduced significantly in *B*. *vulgatus*-transplanted infant mice (Fig. 4d).

### Metabolome profiles revealed the characteristic metabolites that directly modulate intestinal *V*. *cholerae* growth

**Fig. 5:**
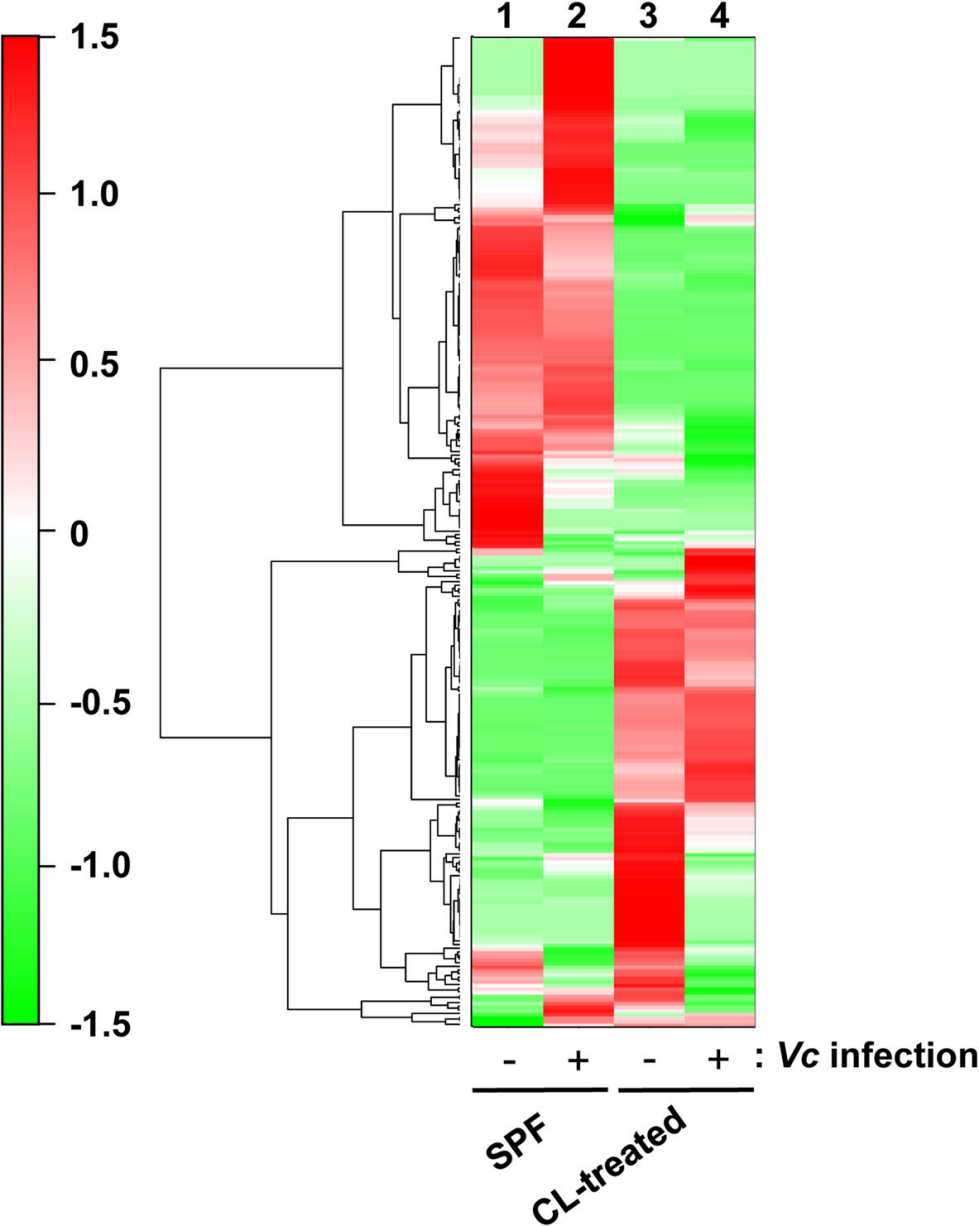
Heat map constructed from hierarchical cluster analysis (HCA) of cecal metabolites. Metabolites (*n* = 273) that exhibit similar detection patterns in four samples are clustered, and the distance between clusters is shown as a tree diagram. The degree of red or green color indicates a larger or smaller amount of a given metabolite. Columns 1 and 2 display metabolome profiles of regular SPF mice, while 3 and 4 shows those of CL-treated groups. Columns 2 and 4 are metabolome profiles after *V*. *cholerae* infection in each group

We next conducted a metabolome analysis to examine whether metabolomic profiles are altered in response to CL treatment and/or *V*. *cholerae* infection. We postulated that alterations of metabolite levels influenced host susceptibility to *V*. *cholerae* infection. Cecal contents were subject to sample preparation for CE-TOF/MS, as described in the “Methods” section. Among the metabolites detected in our analysis, a total of 273 metabolites were successfully quantified and their relative quantities between samples are presented in a heat map (Fig. 5). A table that lists all 273 metabolites and their relative amounts in four samples is shown as supplementary information (a supplementary excel file). Of the many differences between the metabolomic profiles of each group, the most noticeable one is that red and green line clusters representing compounds in large and small quantities, respectively, are generally reversed in control SPF vs. CL-treated group (column 1 vs. 3, Fig. 5). Such striking alterations in metabolome profiles can be interpreted as a consequence of the dramatic microbiota compositional changes, as shown in Fig. 1. Interestingly, a smaller degree of profile change was observed in response to *V*. *cholerae* infection in both experimental groups (column 1 vs. 2 and column 3 vs. 4, Fig. 5). These results demonstrate that (i) CL treatment induces more substantial changes in metabolome profiles than does *V*. *cholerae* infection, and (ii) CL-induced changes on metabolite level might have created an environment that strongly facilitates *V*. *cholerae* growth and pathogenesis.

**Fig. 6:**
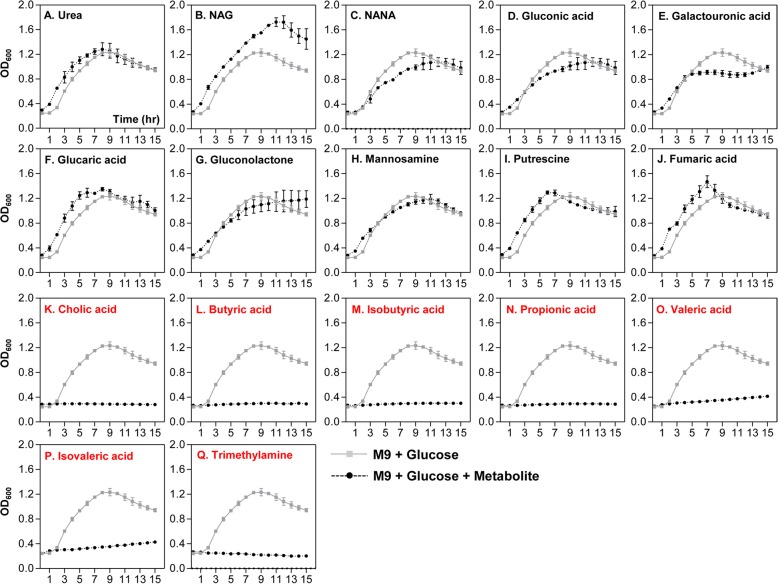
Effects of selected metabolites on the inhibition of *V*. *cholerae* growth in vitro. N16961, a 7th pandemic *V*. *cholerae* strain, was grown in the presence of indicated metabolites in M9 media. A total of 17 metabolites were tested. Medium pH was neutralized by adding NaOH or HCl, when necessary. OD_600_ values were measured to monitor bacterial growth every 2 h. M9 media contains 0.4% glucose as a primary carbon source. Lines in gray show bacterial growth in M9+glucose (M9G), while black lines indicate bacterial growth in M9G supplemented with indicated metabolites. Cholic acid (panel **k**), Butyric acid (panel **l**), Isobutyric acid (panel **m**), Propionic acid (panel **n**), Valeric acid (panel **o**), Isovaleric acid (panel **p**), and Trimethylamine (panel **q**) are highly capable of inhibiting N16961 growth

**Fig. 7:**
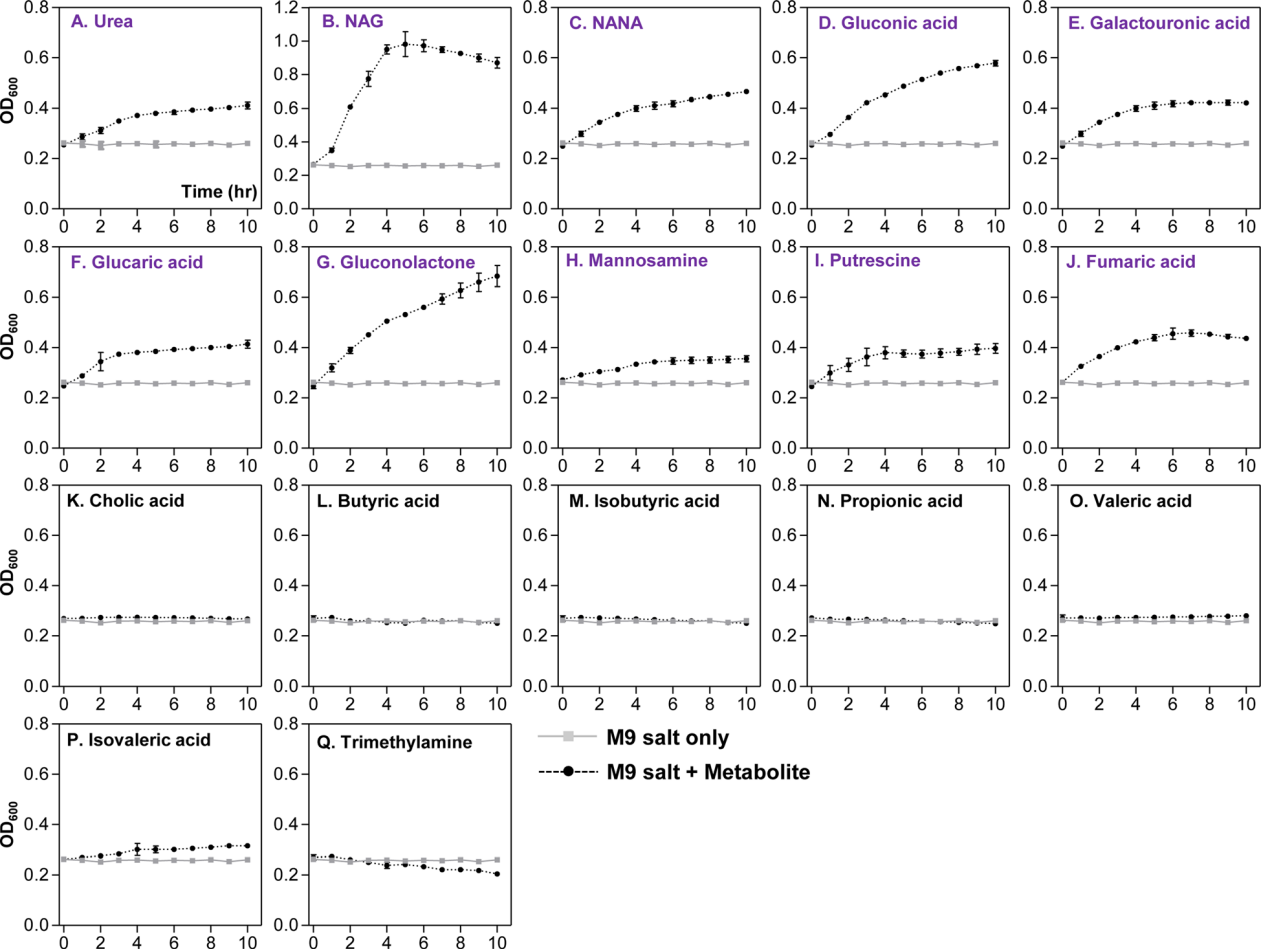
Effects of selected metabolites on the stimulation of *V*. *cholerae* growth in vitro. Bacterial growth was conducted as described in Fig. 6. To examine growth-stimulating capabilities of metabolites, M9 media lacking in glucose was used in experiments. Lines in gray show bacterial growth in M9 with no carbon source, while black lines indicate bacterial growth in M9 supplemented with indicated metabolites. Compounds shown in purple font promoted bacterial growth, albeit to varying degrees. The degree of growth-promoting capability of a given metabolite is displayed semi-qualitatively with “+++”, “++”, or “+” in Table 1. “+++” indicates the metabolite with the strongest growth-promoting capability, while the “+” means that the metabolite induced a mild growth promotion

Table 1 shows a list of metabolites that were detected in the greatest quantities in control SPF (compounds 1~13) or CL-treated (compounds 14~27) mice. Out of the 13 molecules detected in the SPF mouse ceca, all except for cholic acid were not detected in CL-treated mice. It is of particular interest that (iso) butyric acid, propionic acid, and (iso) valeric acid, collectively termed short-chain fatty acid (SCFA) are among the most abundantly detected metabolites in control group (Table 1). Fourteen compounds (14~27), whose relative quantities are greater than 1.0E-03, were selected and presented as dominant metabolites in the CL-treated group (Table 1, bottom portion). Relative quantities of those molecules were either non-detectable (compounds 14, 21, 23, and 27) or considerably smaller in the control SPF group. These results further support the notion that distinct profile change occurred in response to CL treatment. We next examined how *V*. *cholerae* growth is controlled by each of these major compounds. Of the 27 listed compounds, 17 were selected based on the availability and we monitored *V*. *cholerae* in vitro growth in M9 media supplemented with each metabolite. Growth curve experiments shown in Fig. 6 were conducted to reveal compounds that can inhibit *V*. *cholerae* growth. M9 media used in this particular set of growth experiments contain 0.4% glucose as primary carbon source, thereby enabling us to monitor whether the extraneously added metabolite promotes or inhibits *V*. *cholerae* growth. Seven metabolites shown in panels K~Q clearly inhibited *V*. *cholerae* growth. Importantly, these compounds were present in the largest quantities in regular SPF mouse intestines, but either absent or barely detectable in CL-treated mouse intestines (Table 1). Prominent growth promotion was observed when the culture medium included NAG (Fig. 6b), a metabolite detected at sufficiently high level in CL-treated mice. Supplementation with urea (Fig. 6a), glucaric acid (Fig. 6f), putrescine (Fig. 6i), or fumaric acid (Fig. 6j) resulted in a slight growth stimulation, especially during the early stage of growth. Again, these metabolites were detected more abundantly in CL-treated mice, than in the SPF control mice (Table 1). When N16961 growth was assessed in M9 media with an indicated metabolite as the sole carbon source, each metabolite’s ability to stimulate *V*. *cholerae* growth was clearly demonstrated (Fig. 7). Consistent with result shown in Fig. 6b, NAG was most potent in stimulating *V*. *cholerae* growth, with OD_600_ values reaching ~ 1.05 (Fig. 7b). Next to NAG, gluconic acid (Fig. 7d) and gluconolactone (Fig. 7g) were effective at promoting *V*. *cholerae* growth. As expected, seven inhibitory metabolites (cholic acid, butyric acid, isobutyric acid, propionic acid, valeric acid, isovaleric acid, and trimethylamine) still prevented growth of *V*. *cholerae* (Fig. 7k–q). Together, these results illustrate that CL-induced changes in the host intestine are clearly reflected on metabolite level and such major alterations of metabolomic profiles significantly affect *V*. *cholerae* intestinal growth.

**Table 1:**
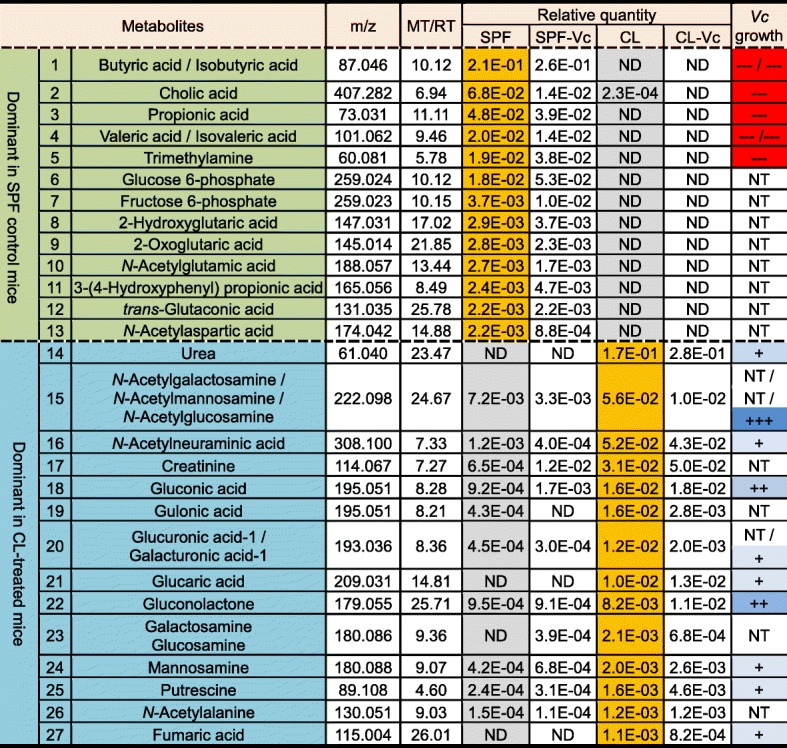
Metabolites found to be dominant in regular SPF and CL-treated mouse intestines and their impacts on *V*. *cholerae* growth

Twenty-seven metabolites that were present exclusively in either group were selected among 273 compounds. Metabolites detected most abundantly in the control group (No.1~13) or in the CL-treated group (No.14~27) are listed in the order of quantity. The growth-inhibiting or growth-promoting capability of a given metabolite is displayed semi-qualitatively with a minus (−) or plus (+) sign, respectively. For example, “+” indicates a metabolite with mild growth-promoting capability, whereas “---” indicates that the metabolite induced strong growth suppression. *ND* not detected, *NT* not tested, *m*/*z* mass-to-charge ratio, *MT* migration time, *RT* retention time, *Vc Vibrio cholerae*. Relative quantity of a metabolite is calculated by the equation: (peak area of the metabolite)/(peak areas of internal standards × sample amount). Peaks are assigned based on the signal-to-noise ratio (S/N) of 3 or above.

### CL-treated adult mice can serve as a model for investigating *V*. *cholerae* pathogenesis

**Fig. 8:**
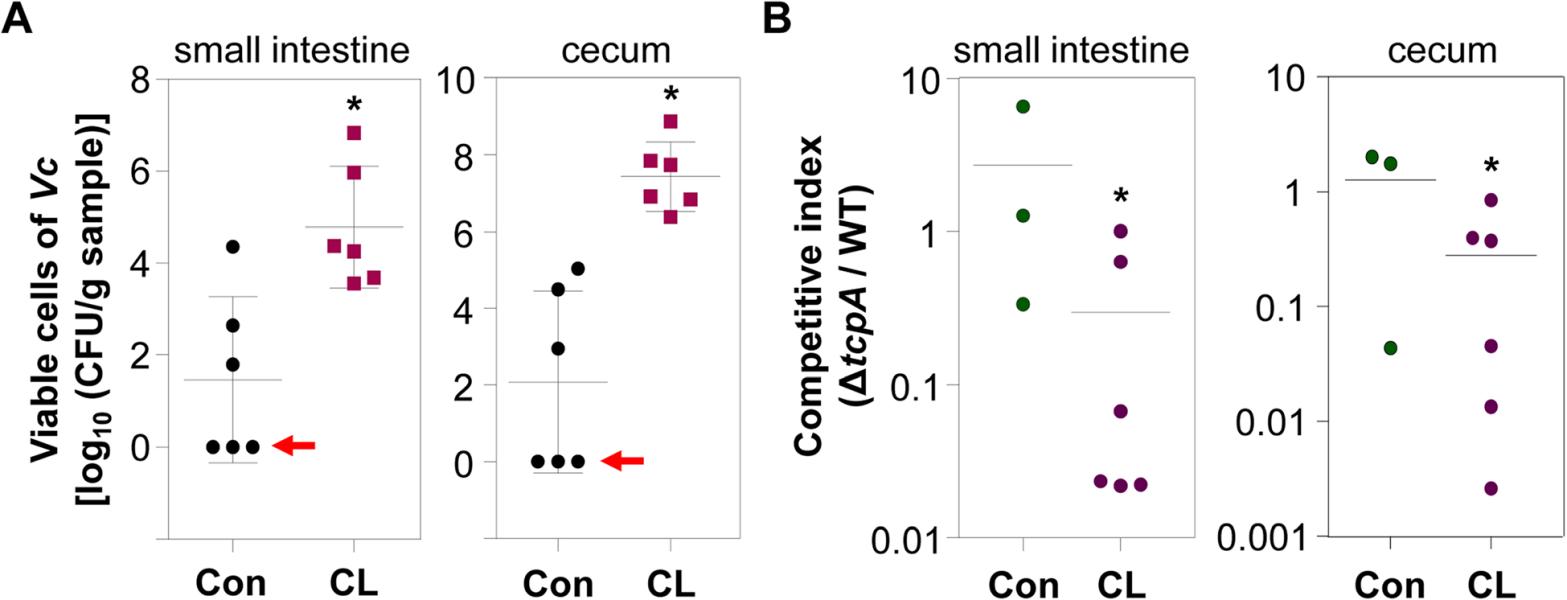
A *V*. *cholerae* Δ*tcpA* mutant is defective in colonizing the intestine of CL-treated adult mice. N16961 and its Δ*tcpA* mutant grown in LB were harvested and mixed at 1:1 ratio. A mixture of 1 × 10^9^ CFU was inoculated via oral gavage into PBS-treated (control) or CL-treated mice (*n* = 6 per group). At 24 h post-infection, mice were sacrificed to measure bacterial colonization. **a** Viable cell numbers of *V*. *cholerae* cells (both N16961 and the Δ*tcpA* mutant). Aliquots of lysates (small intestine or cecum) were serially diluted for *V*. *cholerae* CFU counting on LB agar supplemented with 200 μg/mL SM. Values are displayed on a log scale as mean ± SEM for each group. *P < 0.05 versus bacterial CFUs detected in the control group. In three out of six mice in the control group, no *V*. *cholerae* cells were recovered in the small intestine or cecum (red arrows). **b** The competitive index represents the ratio of Δ*tcpA* mutant to N16961 recovered after infection either in the small intestine or cecum in each mouse after infection. *P < 0.05 versus the competitive index obtained from infection with no CL treatment

Our results demonstrate that infection-induced phenotypes, such as intestinal colonization and fluid accumulation, are clearly observed in CL-pretreated adult mice. Therefore, we finally examined whether or not CL-treated mice can be utilized as a model to delineate *V*. *cholerae* infection. To address this issue, two groups of mice were infected with 1:1 mixture of N16961 and its Δ*tcpA* mutant, a well-characterized *V*. *cholerae* mutant defective in colonizing the intestine [40]. We then compared colonization capabilities of these two strains by calculating the competitive index. In this particular set of experiments, fluid accumulation and bacterial colonization were again substantially increased in CL-treated mice, but not in PBS-treated control group (Additional file 3: Figure S3). *V*. *cholerae* cells were completely eliminated in three out of six mice in control group (red arrow lines, Fig. 8a). Importantly, Δ*tcpA* mutant exhibited significantly reduced colonization capabilities, in comparison to WT N16961 (Fig. 8b). The average values of the competitive indices (Δ*tcpA*/WT) were ~ 0.29 and ~ 0.28 in the small intestine and cecum of CL-treated mice, respectively. In contrast, meaningful differences in colonization capabilities between two strains were not detected, when untreated adult mice were used (Fig. 8b). These results suggest that CL pretreatment not only induces active bacterial colonization but also creates a valuable environment we can exploit to better understand the pathogenesis of *V*. *cholerae*.

## Discussion

A long-standing unresolved question in cholera research is how *V*. *cholerae* has evolved as a human-specific pathogen in nature. It has been of particular interest that GM1 ganglioside, a lipid molecule that binds to CT, is present not only on human intestinal epithelium but also mouse intestinal epithelium [41, 42]. This notion suggests that CT, the most important virulence determinant in *V*. *cholerae*, should also be active in mouse intestine. However, adult mice are naturally resistant to *V*. *cholerae* infection. We hypothesized that understanding compositional differences between human and mouse intestinal microbiotas would help address this important issue of host tropism.

Our 16S rRNA gene sequencing data (Figs. 1 and 3) show that approximately 80% of the C57BL/6 mouse gut microbiota belongs to the Bacteroidetes phylum. Virtually complete elimination of the phylum induced by CL treatment is essential for infecting the murine host with *V*. *cholerae*. However, for our findings to be clinically relevant, it needs to be first established that the mouse gut microbiome is, to a sufficient degree, reflective of the human gut microbiome. Previous studies have reported predominantly high relative abundances of the *Bacteroidetes* phylum in both mouse and human gut microbiomes [43–46]. Furthermore, there is evidence that *B*. *vulgatus* isolates from human and other animal hosts are indistinguishable based on their 16S rRNA gene sequences [47], suggesting phylogenetic proximity between mouse- and human-gut resident *B*. *vulgatus* strains. Nonetheless, the composition of a gut microbiome does not necessarily reflect its functional capacity [46]. Additionally, even though the mouse gut microbiome closely resembles that of humans at the phylum level, the similarity dissipates on lower taxonomic levels, as families and genera that comprise the predominant *Bacteroidetes* phylum are quite different between mice and humans [44]. To compensate for such limitations, we have demonstrated that our key findings are reproduced when GF mice are mono-associated with *B*. *vulgatus* and infected with *V*. *cholerae* (Fig. 4a, b). Given such considerations, it is likely that the relative abundance of *B*. *vulgatus* is an important determining factor for *V*. *cholerae* infectivity not only in murine hosts but in humans as well. An important question would be how abundantly *B*. *vulgatus* cells that share similar functions with mouse isolates are present in human intestine.

Based on our species-level population changes, *Escherichia coli* explosively proliferated during CL treatment and became the most dominant component (Fig. 3). This finding suggests that elevated susceptibility to *V*. *cholerae* infection in CL-pretreated mouse could also be due to the presence of *E*. *coli* cells in large quantity. In line with this notion, an *E*. *coli* strain with robust catalase activity was found to stimulate enhanced *V*. *cholerae* intestinal colonization in neonatal mice [1]. In the same work, increased *V*. *cholerae* colonization occurred in VAN-treated adult mice [1]. In the current study, however, cholera-like symptoms including fluid accumulation in the small intestine and production of watery fecal matter were not observed in the VAN-treated group (Fig. 1k). We therefore conclude that the loss of *B*. *vulgatus* is a much more critical determinant over increased *E*. *coli* for host susceptibility to *V*. *cholerae* infection.

In the present study, we found that CL treatment increases *V*. *cholerae* infectivity in the normally resistant adult mice. Previous studies have reported that antibiotic administration triggers dysbiosis in the gut microbiota [48, 49], and such perturbations lead to suppression of host resistance against various enteropathogenic infections. Since CL treatment results in significant perturbation of the gut microbiome, it is conceivable that this model would demonstrate increased susceptibility to infections by other enteric pathogens such as *Salmonella enterica* serovar Typhimurium, *Citrobacter rodentium*, and *Clostridium difficile.* However, for the aforementioned enteric pathogens, there already exists a well-established adult mouse model [50, 51]. Here, we suggest a simple and convenient animal model to study *V*. *cholerae* infection. The adult mouse model treated with oral CL consistently exhibited cholera-like symptoms. Out of the several already existing animal models for cholera research, the suckling mouse model is considered to be a descriptive model that manifests the cholera-characteristic watery diarrhea. However, the model is not adequate for use in vaccine research, because suckling mice have yet to develop adaptive immunity [52]. Moreover, the susceptibility of suckling mice to *V*. *cholerae* infection might be an artifact caused by their immature immune system, although this idea has not yet been fully verified. Therefore, an infection model using adult mouse has been required for the purpose of unveiling the pathophysiological mechanisms that *V*. *cholerae* employs to induce cholera in the host intestine [53]. However, intestinal fluid secretion and continuous colonization of *V*. *cholerae* do not readily occur in adult mice harboring intact gut microflora [54, 55]. For this reason, the ligated ileal loop model that involves surgical manipulation of the animal’s intestine has been proposed as an alternative [56]. Our CL-induced adult mouse model, which can be established by a simple treatment, clearly reproduces typical cholera symptoms observed in humans, such as the severe watery diarrhea, extensive fluid accumulation, and *V*. *cholerae* colonization within the intestinal mucus layer. Furthermore, in our model, CT has been detected in the intestinal fluid, at a level sufficient to elicit intestinal fluid secretion (Fig. 2c). In addition, the competitive index of Δ*tcpA*/WT showed that TCP, a verified colonization factor of *V*. *cholerae* in human intestine [57, 58], is also essential for the induction of illness in adult mice (Fig. 8). To date, the importance of TCP has been explored only in infant rabbits [53] and infant mice [59].

Host-derived mucus glycan is a nutrient source utilized exclusively by the gut microbiota. Considering the microbial competition for nutritional resources in the intestinal tract and constant release of glycan into the lumen by epithelial cell turnover [60–62], the ability of select microbes to nutritionally exploit the glycans confers a conceivable advantage toward their survival and proliferation. A large proportion of glycan structures consist of *N*-acetyl amino sugars, such as *N*-acetylgalactosamine (GalNAc), *N*-acetylglucosamine (GlcNAc, NAG), and *N*-acetylneuraminic acid (Neu5Ac, NANA). Monosaccharides are produced as the result of mucin glycans being catabolized by the gut microbial species equipped with glycosidase genes. *Bacteroides* has evolved to express glycosidase genes which allow them to perform mucin degradation to full completion, and thus become well-adapted to thrive in the intestine [61–63]. The anaerobes that belong to the Bacteroidetes phylum and *Clostridia* produce pyruvate through glycolysis [61, 64]. Under the anoxic conditions of the intestine, the gut microbiota obtains additional energy by exploiting the pyruvate through anaerobic catabolism. Predominant end products of the anaerobic fermentation are short-chain fatty acids (SCFAs), such as propionic acid, butyric acid, and valeric acid [61].

In this study, the dramatic changes in the cecal metabolomes of CL-treated mice strongly correlate with the compositional shift of the microbiota. Depletion of SCFAs and relatively high occurrence of amino sugars in the ceca of CL-treated mice indicate that catabolism of mucin glycan, a proliferation strategy for microbes belonging to the Bacteroidetes phylum, became inactive upon CL treatment.

Utilization of the host glycan is also important for the invasion of *V*. *cholerae* into the host intestine. When the pathogen enters the intestinal tract, the next strategy for its proliferation is to colonize the epithelial cells covered with a viscous layer of mucus. *V*. *cholerae* effectively responds to the mucus glycan and catabolize the polysaccharides to acquire competitive advantage in host intestine [65–68]. Glucosamine-6-phosphate (GlcNP-6), a common intermediate molecule shared between the catabolic pathways of GlcNAc and Neu5Ac, was reported to be indispensable for *V*. *cholerae* motility [68]. Chemotaxis mediated by motility toward the nutrient sources is important for colonization and proliferation of *V*. *cholerae* [68]. Consistent with this notion, depletion of sialic acid (Neu5Ac) transporters (encoded in *VC1777*-*1779*) attenuates colonization [69]. During this process, *V*. *cholerae* needs to overcome a number of challenges such as acid stress, antimicrobial peptides, host immune response, and SCFAs [64, 66, 68]. The reduction of intestinal SCFAs in patients with diarrhea and recovery of normal SCFA levels after conventional treatment support that the SCFAs could be a contributing factor to eubiosis of the intestinal microbiota [70]. In general, SCFAs are considered to be an effective barrier against pathogenic invasion, although some enteric pathogens exploit SCFAs to sense their surroundings and to decide whether or not to express their arsenal of virulence factors [71].

**Fig. 9:**
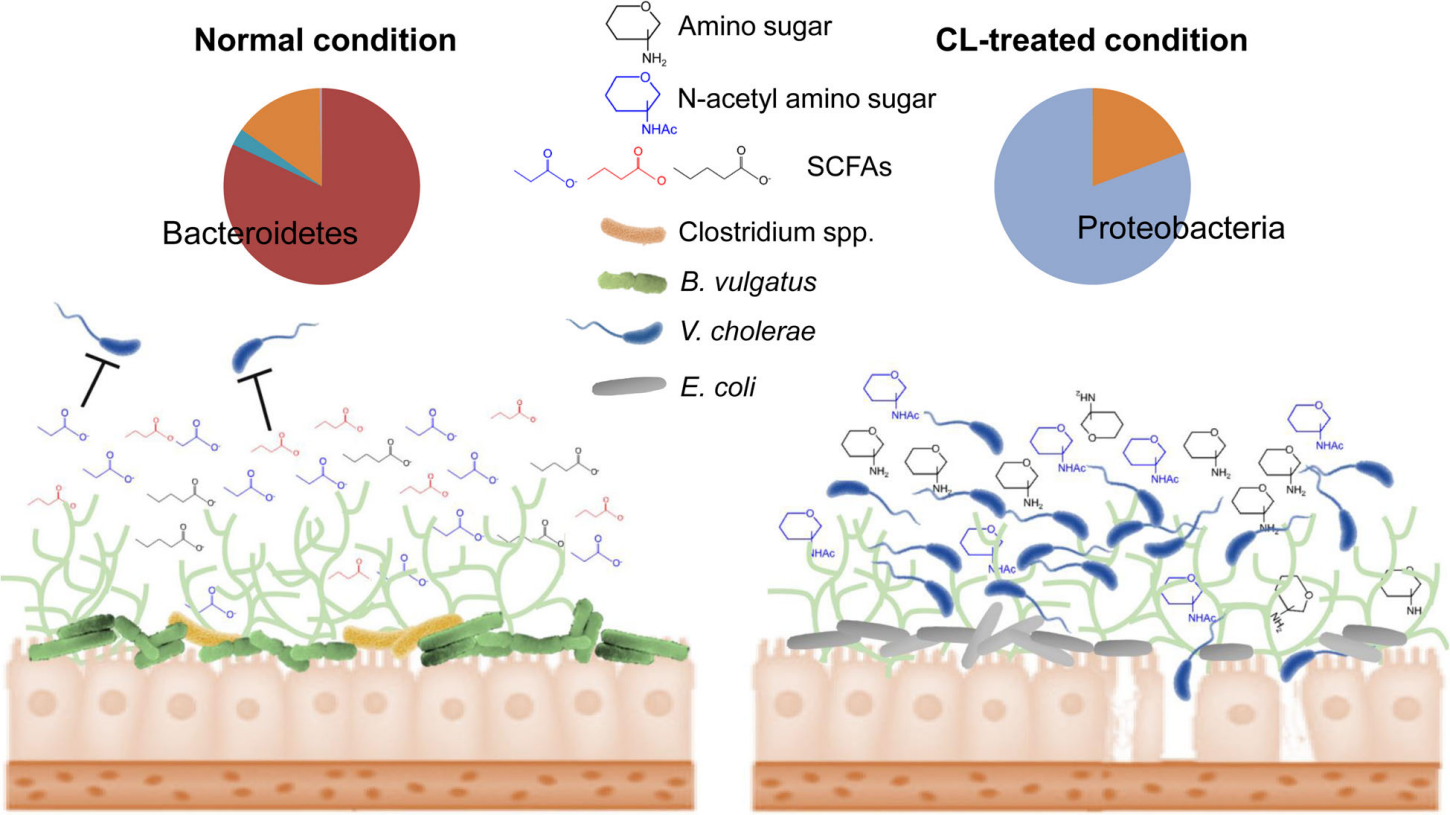
Summary of CL-induced microbiota composition changes and its impact on host susceptibility to *V*. *cholerae* infection. A remarkable shift in commensal microbiota population occurs in response to treatment with CL. Under normal condition, *B*. *vulgatus*, and to a lesser degree, also Clostridium spp. metabolize mucin glycan and accumulation of metabolites including SCFAs that suppress *V*. *cholerae* intestinal growth ensues. Metabolites that increased in quantities upon CL treatment, such as amino sugars, potentiate *V*. *cholerae* intestinal growth

Our results suggest that catabolism of *N*-acetyl amino sugars and subsequent accumulation of SCFAs in the distal intestine are largely managed by specific gut microbiota (Fig. 3). Elimination of the anaerobic *Bacteroides* and *Clostridium* genera caused by CL treatment results in drastic changes of intestinal metabolites as summarized in Fig. 9. Especially, propionic and butyric acid mainly produced by *Bacteroides* and *Clostridium*, respectively, are dramatically reduced after CL treatment, thus leaving a variety of metabolites (NAG, NANA, gluconate, gluconolactone, etc.) available for utilization by *V*. *cholerae* (Fig. 9). Of particular note, gluconate and its intermediate metabolites were found to enhance the colonization and virulence of *V*. *cholerae* within the intestine [72]. In the perturbed intestinal environment, *V*. *cholerae* readily acquires nutrient sources as there is no competition with the host glycan-degrading and thus SCFA-producing gut microbiota (mainly *Bacteroides* and *Clostridium*) genera.

## Conclusions

In conclusion, we identified a commensal bacterial species, *B*. *vulgatus*, that plays a significant role in inhibiting *V*. *cholerae* colonization in the adult mouse intestine. Moreover, we propose a simple antibiotic-treated mouse model to delineate *V*. *cholerae* infection, which might prove a useful tool for cholera vaccine research. We hope that the results presented in the present study will stimulate future investigations to better understand the in vivo probiotic function of *B*. *vulgatus* against *V*. *cholerae* infection and to devise better strategies for tackling this virulent and clinically important human pathogen.

## Methods

### Bacterial strains and growth condition

*Vibrio cholerae* O1 serotype N16961 was used as a model pathogen in all experiments [73]. N16961 was routinely grown in Luria-Bertani (LB) broth (10 g NaCl, 10 g tryptone, and 5 g yeast extract per L) or on LB agar plates (15 g agar per L) at 37 °C under aerobic condition. In order to selectively isolate N16961 from feces, SI, cecum, and colon, we used streptomycin (Duchefa) at a 200 μg/mL concentration. Another selective medium used to isolate *V*. *cholerae* from intestinal contents was thiosulfate-citrate-bile salt-sucrose (TCBS) medium. For enumeration of intestinal *V*. *cholerae* loads, mouse intestines were manually extracted from infected animals. The SIs, ceca, and colons were then homogenized in PBS (Sigma-Aldrich) and centrifuged at 1000 rpm for 1 min. The supernatants were serially diluted in PBS, and spotted on an LB agar plate containing streptomycin. *B*. *vulgatus* strains were isolated from C57BL/6 mouse intestines using Bacteroides Bile Esculin (KisanBio, Korea) agar plates. Subsequently, *B*. *vulgatus* strains were cultivated on Gifu Anaerobic Media (GAM) broth (KisanBio, Korea) in an anaerobic chamber maintained at 90% N_2_, 5% CO_2_, and 5% H_2_. All anaerobic culture media were deoxygenated for at least 24 h prior to usage. RAPD analysis was performed using six different *B*. *vulgatus* colonies recovered independently from two different CL-treated mice, following procedures described elsewhere [1].

### Mouse husbandry and antibiotic treatment

Experiments were performed using both SPF and GF mice in a C57BL/6 genetic background. SPF mice were obtained from Orient Bio (Sungnam, Korea), and GF mice were generated by the Yonsei University College of Medicine GF mouse facility. Mice were provided sterile water and autoclaved food. GF status was confirmed by negative microbial growth in mouse feces. GF mice were bred and housed in flexible-film isolators (Class Biologically Clean Ltd., Madison, WI). When necessary, GF mice were transferred to isocages (Tecniplast Inc., Italy) for experimental grouping. All mouse experiments were conducted according to the guidelines provided by the Department of Animal Resources of Yonsei Biomedical Research Institute. The Committee on the Ethics of Animal Experiments at Yonsei University College of Medicine approved this study (Permit numbers, 2017-0210 and 2018-0250).

For the antibiotic treatment presented in Fig. 1, 8- to 9-week-old C57BL/6 female mice were administered either SM (1 mg), VAN (250 μg), and CL (1 mg), or PBS by oral gavage once a day for 5 days. The dosage of the antibiotics was chosen to induce dysbiosis but not complete elimination of commensal species of the gut microbiota. Each oral gavage treatment did not exceed 100 μl. For the antibiotic treatment shown in Additional file 2: Figure S2, mice were treated with a single dose of CL (10 mg).

### *V*. *cholerae* infection and GF mouse manipulation

*V*. *cholerae* cells grown aerobically overnight at 37 °C were diluted 100-fold to inoculate fresh LB broth medium. The sub-cultured broth was incubated in a shaking incubator at 37 °C for 4 h. Finally, *V*. *cholerae* cells were centrifuged at 13,000 rpm for 10 min, and the resulting pellet was suspended in sterile PBS to the concentration of 10^10^ CFU per mL. For infection, 8- to 9-week-old mice were orally infected with 50 μl of *V*. *cholerae* cell suspension containing approximately 5 × 10^8^ CFU of *V*. *cholerae* cells. The fluid accumulation ratio was calculated by using the equation (intestine weight) / (total body weight-intestine weight). CT level was quantified by CT ELISA [38]. To enumerate *V*. *cholerae* cells in mouse feces, fecal pellets were physically disrupted to homogeneity, and fecal suspensions were serially diluted for CFU counting. Shortly after euthanasia, SI, cecum, and colon samples were manually extracted from each mouse and homogenized in 5 mL of PBS. The sample homogenates were centrifuged at low speed for remnant tissue removal, and the resulting supernatant was used for CFU counting.

FMT was performed to transplant the antibiotic-treated gut microbiota into GF mice. SPF mice were treated either with CL or PBS as per the procedure described above, and fresh feces were collected 24 h post-antibiotic treatment. In each antibiotic-treated and control group, there were three mice, and two fecal pellets were collected from each mouse. The fecal pellets collected were pooled per group and suspended in 600 μl of PBS. The suspension was quick-centrifuged to remove the debris, and the resulting supernatants were orally administered. Each GF mouse received 50 μl of FMT solution by oral gavage. For *B*. *vulgatus* mono-association, *B*. *vulgatus* was first seeded with thawed stock and passaged at least twice and less than 4 times for maximum viability. *B*. *vulgatus* cells were grown in GAM broth under anaerobic conditions. The cultured *B*. *vulgatus* cells were resuspended in PBS, and 10^8^ CFU cells were delivered orogastrically to GF mice. Infant mouse infection was performed following procedures described previously [39].

### Competitive index assay

To proceed with the competitive index assay, *lacZ*-negative wild-type strain N16961 and *lacZ*-positive *ΔtcpA* were cultured as per the aforementioned *V*. *cholerae* culture protocol. Sub-cultured wild-type and *ΔtcpA* cells were centrifuged at 13,000 rpm for 2 min and then resuspended in PBS. Each strain was mixed at a 1:1 ratio, and 100 μl of the prepared mixture (total 10^9^ CFU) was administered to 8- to 9-week-old female C57BL/6 mice (either untreated or CL-treated) by oral gavage. Each mouse was infected with 5 × 10^8^ CFU of each strain.

### Amplicon sequencing for microbiome profiling

The extraction method for bacterial DNA was performed using a PowerMax Soil DNA Isolation Kit (MO BIO). Each sequenced sample was prepared according to the Illumina 16S Metagenomic Sequencing Library protocols to amplify the V3 and V4 regions (519F-806R). The DNA quality was measured by PicoGreen and Nanodrop. Input gDNA (10 ng) was PCR amplified. The barcoded fusion primer sequences used for amplifications were as follows: 341F: 5′-CCTACGGGNGGCWGCAG, and 806R: 5′-GACTACHVGGGTATCTAATCC. The final purified product was then quantified using qPCR according to the qPCR Quantification Protocol Guide (KAPA Library Quantification kits for Illumina Sequencing platforms) and qualified using a LabChip GX HT DNA High Sensitivity Kit (PerkinElmer, MA, USA). Paired-end (2 × 300 bp) sequencing was performed by Macrogen using the MiSeq™ platform (Illumina, San Diego, CA, USA). For species identification, sequenced reads were aligned using blast and NCBI 16S microbial database (version downloaded 2018.01.14). Taxonomy information was assigned only to OTUs that met both query coverage > 85% and identity percent > 85%. OTUs that did not meet these two criteria were left unassigned. In general, microbial species was identified with ~ 99% identity with a reference sequence.

### Metabolite extraction

For extracting ionic metabolites, approximately 50 mg of cecal contents was dissolved in MilliQ water containing internal standards (H3304-1002, Human Metabolome Technologies (HMT), Tsuruoka, Yamagata, Japan) at a ratio of 1:9 (w/v). After centrifugation, the supernatant was centrifugally filtered through a Millipore 5000-Da cutoff filter (UltrafreeMC-PLHCC, HMT) to remove macromolecules (9100×*g*, 4 °C, 60 min) for subsequent analysis with capillary electrophoresis time-of-flight mass spectrometry (CE-TOFMS).

### Metabolome analysis

Metabolome analysis was conducted at HMT using the Basic Scan package with CE-TOFMS for ionic metabolites, based on the methods described previously [74, 75]. Briefly, CE-TOFMS analysis was carried out using an Agilent CE capillary electrophoresis system equipped with an Agilent 6210 time-of-flight mass spectrometer, Agilent 1100 isocratic HPLC pump, Agilent G1603A CE-MS adapter kit, and Agilent G1607A CE-ESI-MS sprayer kit (Agilent Technologies, Waldbronn, Germany). The systems were controlled by Agilent G2201AA ChemStation software version B.03.01 for CE (Agilent Technologies) and connected by a fused silica capillary (50 μm *i.d.*×80 cm total length) with commercial electrophoresis buffer (H3301-1001 and H3302-1021 for cation and anion analyses, respectively, HMT) as the electrolyte. The spectrometer was scanned from *m*/*z* 50 to 1000 [74]. Peaks were extracted using the automatic integration software MasterHands (Keio University, Tsuruoka, Yamagata, Japan) to obtain peak information including *m*/*z*, peak area, and migration time (MT) for CE-TOFMS analysis [76]. Signal peaks corresponding to isotopomers, adduct ions, and other product ions of known metabolites were excluded, and remaining peaks were annotated according to the HMT metabolite database based on *m*/*z* values with the MTs determined by TOFMS. Areas of the annotated peaks were then normalized based on internal standard levels and sample amounts to obtain relative levels of each metabolite. Hierarchical cluster analysis (HCA) was performed using the proprietary software supplied by HMT, PeakStat.

### *V*. *cholerae in vitro* growth curve experiments

N16961 was grown in M9 media supplemented with 17 metabolites at 20 mM each, with the exception of *N*-acetylglucosamine (GluNAc), *N*-acetylneuraminic acid (NANA), glucaric acid, gluconolactone, mannosamine, and cholic acid. These metabolites were added at concentrations of 4, 1, 4, 10, 1, and 0.2 mM, respectively. N16961 growth was monitored by measuring OD_600_ values spectrophotometrically. To examine growth-inhibiting capabilities of the metabolites, N16961 was grown in M9 plus 0.4% glucose. However, M9 salt without glucose was used to assess the capabilities of the metabolites to promote the growth of *V*. *cholerae*.

### Statistical analysis

Data are expressed as mean ± standard error of the mean (SEM). Unpaired Student’s *t* test was used to determine whether differences between groups were significant. A *p* value < 0.05 was considered to indicate statistical significance. All experiments were repeated for reproducibility.

## Supplementary information

**Additional file 1: Figure S1.** Effects of a single-dose treatment of CL on host resistance to *V. cholerae* infection.

**Additional file 2: Figure S2.** Random Amplified Polymorphic DNA (RAPD) analysis of *B. vulgatus* clones.

**Additional file 3: Figure S3.** A *V. cholerae* Δ*tcpA* mutant is defective in colonizing the intestine of CL-treated adult mouse.

## Data Availability

Unprocessed amplicon sequencing results (shown in Fig. 1 and Additional file 1: Figure S1) are provided in the supplementary spreadsheet. In addition, a complete list of metabolites and their relative quantities are displayed in the supplementary spreadsheet. Additional data will become available upon request.

## Abbreviations

CL: Clindamycin
CT: Cholera toxin
FA: Fluid accumulation
FMT: Fecal microbiota transplantation
GalNAc: *N*-acetylgalactosamine
GAM: Gifu anaerobic media
GF: Germ-free
HCA: Hierarchical cluster analysis
LB: Luria-Bertani
NAG, GlcNAc: *N*-acetyl glucosamine
NANA: *N*-acetylneuraminic acid
SCFA: Short-chain fatty acids
SM: Streptomycin
SPF: Specific pathogen free
TCBS: Thiosulfate-citrate-bile salt-sucrose
TCP: Toxin-coregulated pilus
VAN: Vancomycin
