# Supplementary material for: Commensal-derived metabolites govern Vibrio cholerae pathogenesis in host intestine
Source: Microbiome. 2019 Sep 14;7:132. doi: 10.1186/s40168-019-0746-y (PMC6744661; doi:10.1186/s40168-019-0746-y)
Supplement: Supplementary file 1 — Additional file 1: Figure S1. Effects of a single-dose treatment of CL on host resistance to V. cholerae infection. [file 40168_2019_746_MOESM1_ESM.docx]

**
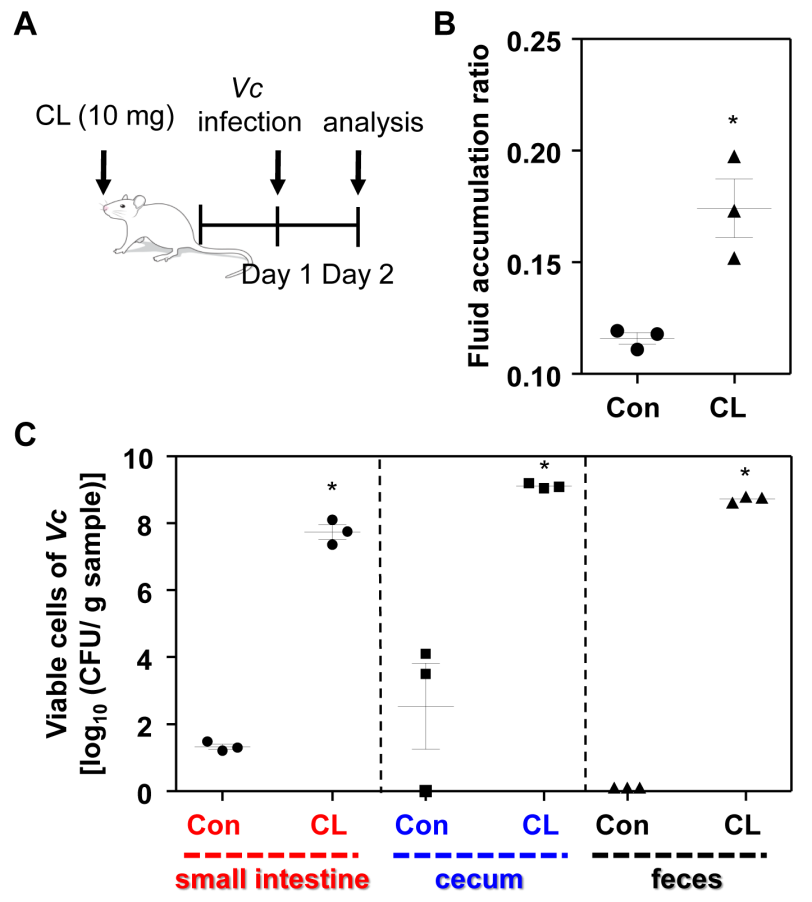
**

**Figure S1. Effects of a single-dose treatment of CL on host resistance to *V. cholerae* infection.** (**A**) Schematic diagram of the experimental procedure. C57BL/6 (8~9 weeks old) mice were treated by oral gavage with CL (10 mg/Kg) or PBS as a control. At 24 hr post-treatment, mice were challenged with *V. cholerae* infection (5X10^8^ CFU) for 24 hours. (**B**) At the end of the infection period, fluid accumulation ratio was calculated by the equation of (intestine weight)/[(total body weight)-(intestine weight)]. **P*<0.05 versus the control group. (**C**) Lysates of small intestine or cecum were prepared by tissue homogenization. Fecal suspensions from each mouse were also prepared by physical grinding. Aliquots of lysates or suspensions were serially diluted for *V. cholerae* CFU counting on LB agar supplemented with 200 μg/mL SM. Values are expressed as means ± SEM in each treatment group and displayed on a log scale. **P*<0.001 versus bacterial CFUs detected in the control group.
