# Supplementary material for: Commensal-derived metabolites govern Vibrio cholerae pathogenesis in host intestine
Source: Microbiome. 2019 Sep 14;7:132. doi: 10.1186/s40168-019-0746-y (PMC6744661; doi:10.1186/s40168-019-0746-y)
Supplement: Supplementary file 2 — Additional file 2: Figure S2. Random Amplified Polymorphic DNA (RAPD) analysis of B. vulgatus clones. [file 40168_2019_746_MOESM2_ESM.docx]

**
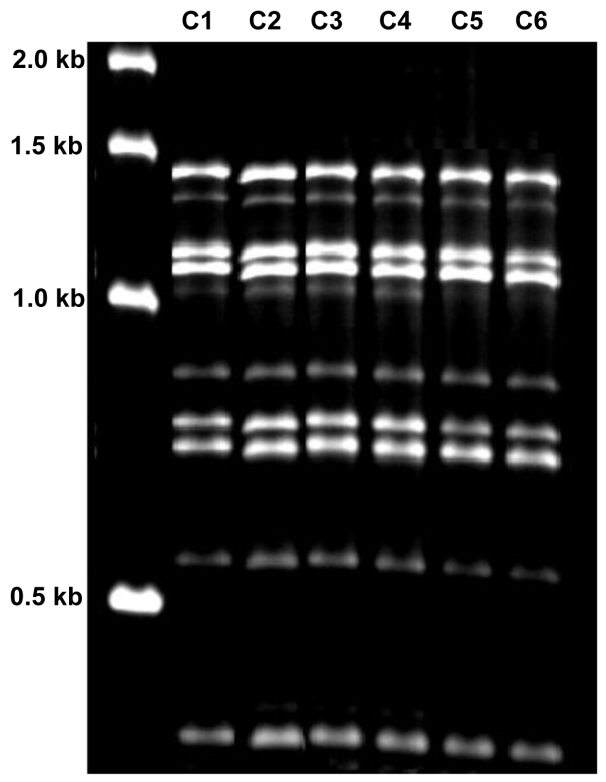
**

**Figure S2. Random Amplified Polymorphic DNA (RAPD) analysis of *B. vulgatus* clones.**  The RAPD amplification reaction was performed as described in Materials and Methods. Reaction products were analyzed on agarose gels with molecular weight ladders on the far-left lane. A total of 6 colonies isolated from mouse feces were used for the analysis. All of the clones produced identical amplification products.
