# Supplementary material for: Commensal-derived metabolites govern Vibrio cholerae pathogenesis in host intestine
Source: Microbiome. 2019 Sep 14;7:132. doi: 10.1186/s40168-019-0746-y (PMC6744661; doi:10.1186/s40168-019-0746-y)
Supplement: Supplementary file 3 — Additional file 3: Figure S3. A V. cholerae ΔtcpA mutant is defective in colonizing the intestine of CL-treated adult mouse. [file 40168_2019_746_MOESM3_ESM.docx]

**
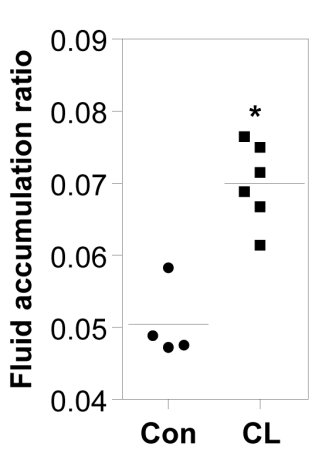
**

**Figure S3. A *V. cholerae* Δ*tcpA* mutant is defective in colonizing the intestine of CL-treated adult mouse.** N16961 and its Δ*tcpA* mutant grown in LB were harvested and mixed at 1:1 ratio. A mixture of 2X10^8^ CFU was inoculated via oral gavage into PBS-treated (control, n=4) or CL-treated mice (n=6). At 24 hr post-infection, mice were sacrificed to measure fluid accumulation ratio as described in Fig. S3B. **P*<0.05 versus the control group.
